# Association Between Coeliac Disease and Risk of Any Malignancy and Gastrointestinal Malignancy: A Meta-Analysis

**DOI:** 10.1097/MD.0000000000001612

**Published:** 2015-09-25

**Authors:** Yuehua Han, Wuzhen Chen, Peiwei Li, Jun Ye

**Affiliations:** From the Department of Gastroenterology (YH, WC, PL, JY), The Second Affiliated Hospital, Zhejiang University School of Medicine; and Cancer Institute (Key Laboratory of Cancer Prevention and Intervention, China National Ministry of Education, Key Laboratory of Molecular Biology in Medical Sciences, Zhejiang Province, China), The Second Affiliated Hospital (JY), Zhejiang University School of Medicine, Hangzhou, Zhejiang Province, China.

## Abstract

Coeliac disease (CD) is reported to be associated with risk of malignancy; however, this association remains unclear. We aimed to systematically evaluate the association between CD and risk of all malignancies as well as gastrointestinal (GI) malignancy specifically.

The PUBMED and EMBASE databases were searched to identify eligible studies from 1960 to March 2015, without restriction. Two reviewers independently performed the study inclusion and data extraction methods. Odds ratios (ORs), risk ratios, or standardized incidence ratios were pooled using either a fixed- or a random-effects model. Sensitivity and subgroup analyses were used to explore sources of heterogeneity.

A total of 17 studies were included in this meta-analysis. The pooled OR for risk of all malignancies was 1.25 (95% confidence interval [CI] 1.09–1.44), whereas the pooled OR for risk of GI malignancy was 1.60 (95% CI 1.39–1.84) and suggested an inverse association with CD. Moreover, patients with CD were at a higher risk of esophageal cancer (pooled OR = 3.72, 95% CI 1.90–7.28) and small intestinal carcinoma (pooled OR = 14.41, 95% CI 5.53–37.60), whereas no significant associations were observed for other GI cancers, including gastric, colorectal, liver, and pancreatic cancers. Subgroup analyses also indicated that the results were influenced by the CD diagnostic method, as well as the follow-up time after CD diagnosis.

CD was associated with increased risk of all malignancies as well as GI malignancies, including esophageal cancer and small intestinal carcinoma.

## Abstract 2

Supplemental Digital Content is available in the text

## INTRODUCTION

Coeliac disease (CD) is a chronic autoimmune enteropathy that occurs in 1% of the Western population.^1^ CD is triggered by the ingestion of gluten, which exists in wheat, rye, and barley, and is characterized by small intestinal mucosal inflammation and villous atrophy (VA).^2^ Currently, the only treatment for CD is a gluten-free diet.^3^

The association between CD and malignancy risk has been evaluated previously.^4^ A population-based study revealed a 100-fold increased risk of non-Hodgkin lymphoma in CD patients in the 1960s.^5^ In recent years, a growing number of studies reported a 6- to 9-fold higher incidence of enteropathy-associated T-cell lymphoma and non-Hodgkin neoplasm among CD patients compared with the general population.^4,6^ With regard to gastrointestinal (GI) neoplasms, it was noted that CD patients have a higher risk of developing small bowel adenocarcinoma, with an estimated odds ratio (OR) ranging from 4.29 to 59.97.^7^ In 2002, Askling et al^8^ demonstrated increased colon carcinoma risk (standardized incidence ratio, SIR = 1.9, 95% confidence interval [CI] 1.2–2.8) in CD patients in a population-based cohort, and the carcinomas occurred mainly in the ascending and transverse colon. However, in 2014, Volta et al^9^ indicated that CD has a protective effect against colon cancer (SIR = 0.29, 95% CI 0.07–0.45). Moreover, studies assessing the association between CD and risks of liver and pancreatic cancers were inconclusive and produced mixed results, whereas association with risk of other common malignancies has been reported. Evidence suggests that CD is associated with a reduced risk of breast cancer (hazard ratio [HR] = 0.70, 95% CI 0.62–0.79).^7^ Meanwhile, there is no evidence that CD patients have a higher risk of other malignancies, including lung, prostate, and thyroid cancers.^10–12^

Considering these uncertainties, we performed a systematic analysis aiming to clarify the risk of malignancies in CD patients.

## METHODS

### Literature Search and Study Selection

This meta-analysis was designed, conducted, and reported according to the PRISMA statement.^13^ As this meta-analysis did not involve animal experiments or direct human trials, ethics review board approval and patient consent were not required.

We conducted a comprehensive literature search in the PUBMED and EMBASE databases from 1960 to March 2015. The following terms were used in the search procedure (“coeliac disease” OR “coeliac disease”) AND (“cancer” OR “tumor” OR “carcinoma” OR “neoplasm”). Reference lists of relevant articles and reviews were also searched for possible studies. No language restrictions were applied in the literature search or the study selection. We carefully examined the retrieved studies to exclude potential duplicates or overlapping data. For the articles selected from the literature search, titles and abstracts were first scanned for potential inclusion, and full articles were reviewed subsequently to determine inclusion of eligible studies.

Criteria for inclusion were as follows: a cohort or case-control study; the study assessed the association between CD and risk of all cancers or GI cancer; and the study reported the risk estimate as an OR, SIR, relative risk (RR), or HR with a 95% CI. Compliance to a gluten-free diet may be a confounding factor for malignancy risk in CD patients; however, most studies did not report the effects of a gluten-free diet on the risk of all malignancies or GI malignancy. Thus, we included studies regardless of compliance to a gluten-free diet, and both controlled and uncontrolled CD patients were included in this meta-analysis.

### Data Extraction

Two reviewers performed the data extraction using standardized forms, and discrepancies were resolved by discussion or by a third investigator. The following information was extracted from each study: first author, publication year, study design, sample size of the study, sex and age of participants, country of origin, years of follow-up, method of CD diagnosis, and risk estimates. Ratios that reflected the greatest degree of control for potential confounders were used.

### Statistical Analysis

Heterogeneity across individual studies was evaluated by the χ^2^ test and the I^2^ test, and *P* ≤ 0.05 and/or I^2^ > 50% indicated significant heterogeneity, respectively.^14^ Study-specific OR and RR estimates for CD and cancer risk were pooled using a random-effects model if there was significant heterogeneity; otherwise, a fixed-effects model was applied. Sensitivity and subgroup analyses were performed to explore the source of heterogeneity in the analyses of all cancer and GI cancer risks. Subgroup analyses were also performed in the analysis of esophageal and small intestinal cancer, as CD was significantly associated with the risks of these 2 cancers. Begg funnel plots and Egger test were used to assess publication bias. All analyses were conducted using Stata software (version 11.0; StatCorp, College Station, TX). A *P* value <0.05 was considered statistically significant.

## RESULTS

### Study Inclusion and Characteristics

**FIGURE 1:**
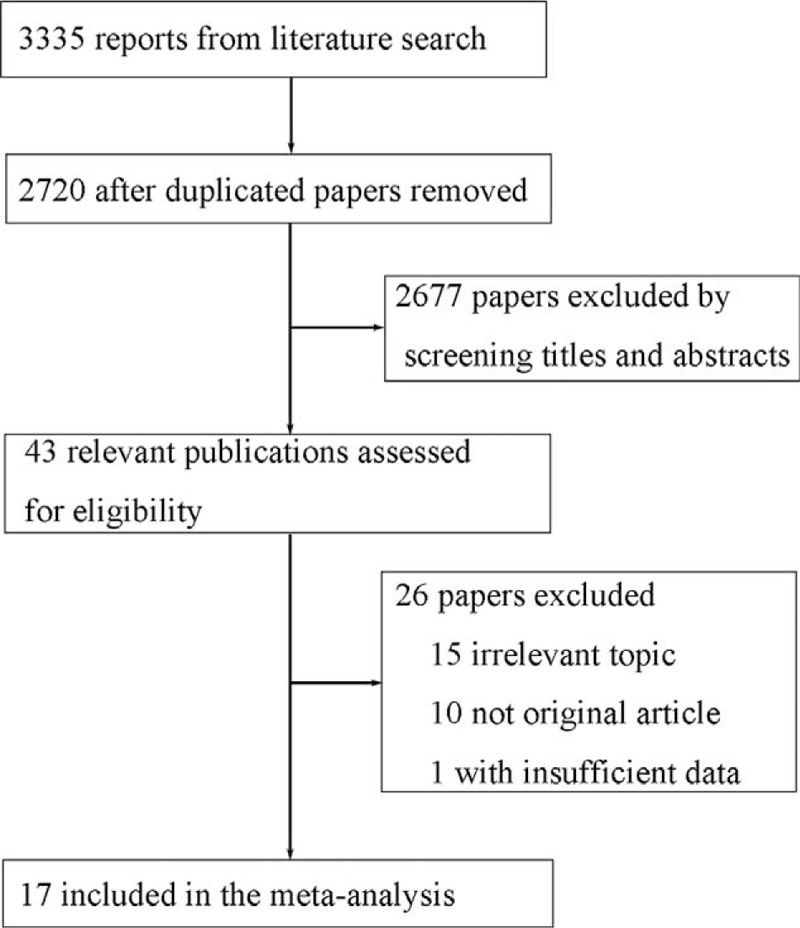
Flow diagram of the study selection process.

A literature search of PUBMED and EMBASE databases yielded 3335 reports, and 2720 were scanned for potential eligibility after removing duplicate publications. Finally, 17 articles were included in this meta-analysis.^4,7–9,15–27^Figure 1 shows the selection process, whereas Supplementary Table 1, http://links.lww.com/MD/A424, indicates the characteristics of the included studies. Among the included studies, 14 reported an association between CD and the risk of all cancers, whereas 15 evaluated the risk of GI cancers.

### Association Between CD and Risk of All Cancers

**FIGURE 2:**
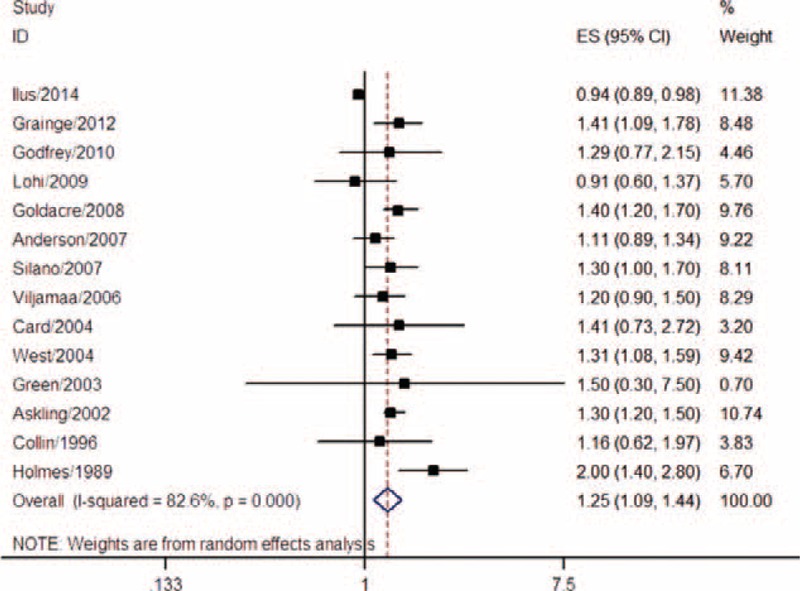
Meta-analysis of the association between coeliac disease and risk of all cancers.

A total of 14 studies evaluated the association between CD and the risk of all cancers. These studies comprised 55,504 CD patients, of whom 2558 developed cancer. All included studies were conducted in Western countries: 5 in Northern Europe, 8 in other European countries, and 1 in the United States. Among the included studies, 13 were cohort studies (6 prospective and 7 retrospective), and the other was a nested case-control study. Three studies used an internal comparison, whereas the others used national data from the same country for external comparison. For CD diagnosis, 6 studies used medical records, 5 studies used histopathology, and 3 studies applied serology methods for latent or undiagnosed CD. Pooled analysis resulted in an OR of 1.25 (95% CI 1.09–1.44) with significant heterogeneity (I^2^ = 82.6%, *P* < 0.001) (Table 1, Figure 2), indicating that CD was associated with an increased risk of all cancers.

**TABLE 1:**
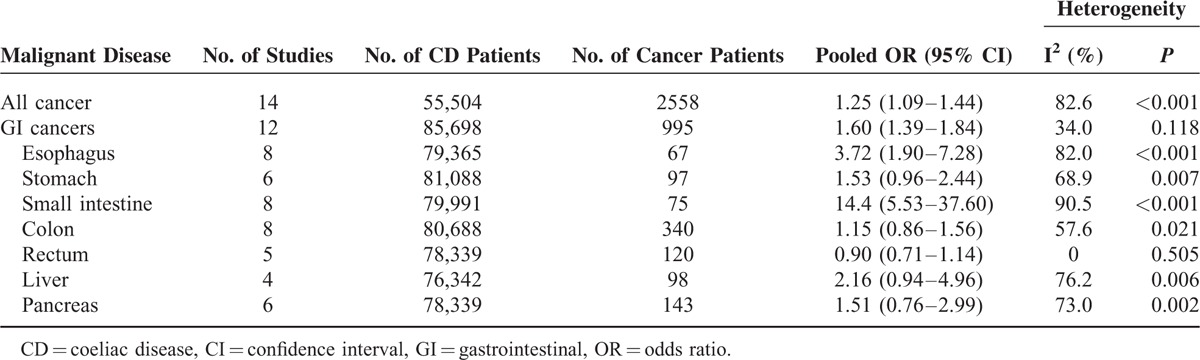
Risk of Cancer in Coeliac Disease

Subgroup analyses revealed that CD patients had a higher risk of all cancers both in prospective (pooled OR = 1.20, 95% CI 1.02–1.40) and retrospective studies (pooled OR = 1.29, 95% CI 1.08–1.54). Moreover, a significant association was observed both in studies that used an internal comparison (pooled OR = 1.23, 95% CI 1.04–1.46) and in studies that used routine data for external comparison (pooled OR = 1.28, 95% CI 1.09–1.50). As reported, risk of all cancers in CD patients might be influenced by the time of diagnosis, as the ORs were different for peridiagnosis and postdiagnosis periods. A total of 4 studies evaluated the peridiagnosis period (within 4 years after diagnosis: Grainge et al^21^; within 2 years: Ilus et al^7^ and Card et al^16^; and within 1 year: West et al^27^), whereas 7 studies evaluated the postdiagnosis period (at least 4 years after diagnosis: Grainge et al^21^; 2 years: Ilus et al^7^ and Card et al^16^; and 1 year: Goldacre et al,^20^ Anderson et al,^15^ Card et al,^16^ and Askling et al^8^). The pooled results suggested that CD increased the risk of all cancers in the peridiagnosis period (pooled OR = 1.72, 95% CI 1.14–2.59), but not in the postdiagnosis period (pooled OR = 1.09, 95% CI 0.93–1.27). The subgroup analysis results for CD diagnostic method, sample size, and geographic region are shown in Table 2. Heterogeneity across studies was influenced by study design, comparison population, CD diagnostic method, sample size, and geographic region (Table 2).

**TABLE 2:**
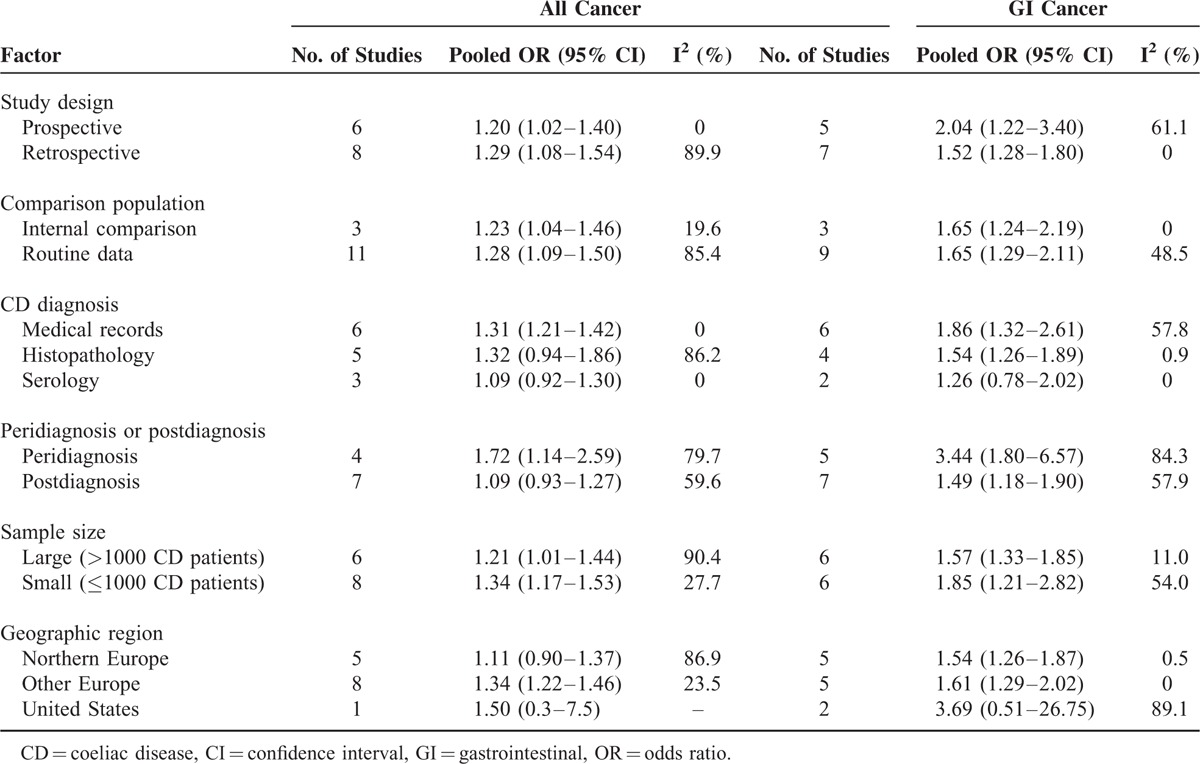
Subgroup Analyses of Association Between Coeliac Disease and Risk of All Cancer and GI Cancer

### Association Between CD and GI Cancer Risk

**FIGURE 3:**
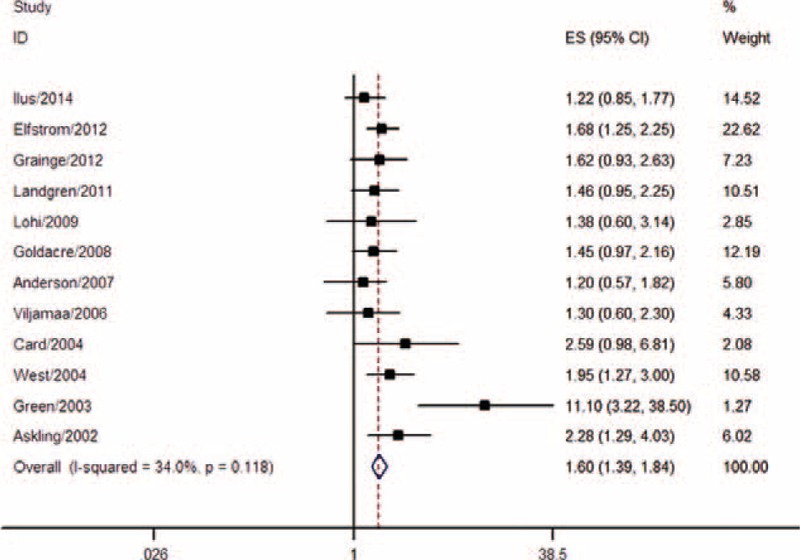
Meta-analysis of the association between coeliac disease and risk of GI cancer. GI = gastrointestinal.

Twelve studies comprising 85,698 CD patients and 995 GI cancers were included. Among these, 5 studies were conducted in Northern Europe, 5 in the United Kingdom, and 2 in the United States. All studies were cohorts, including 5 prospective and 7 retrospective studies. Internal comparison was used in 3 studies, whereas the other 9 adopted external comparisons. For CD diagnosis, 6 studies used medical records, 4 studies used histopathology, and 2 studies applied serology methods for latent or undiagnosed CD. The pooled results demonstrated that CD was associated with a 60% increase in GI cancer risk (pooled OR = 1.60, 95% CI 1.39–1.84) (Table 1, Figure 3). No significant heterogeneity was found (I^2^ = 34.0%, *P* = 0.118) (Figure 3).

As the results of subgroup analyses indicated, a significant association was observed both in the prospective cohort studies (pooled OR = 2.04, 95% CI 1.22–3.40) and in retrospective studies (pooled OR = 1.52, 95% CI 1.28–1.80), as well as in studies using both internal comparisons (pooled OR = 1.65, 95% CI 1.24–2.19) and external comparisons (pooled OR = 1.65, 95% CI 1.29–2.11). Risk of GI cancer in CD patients during the postdiagnosis period (pooled OR = 1.49, 95% CI 1.18–1.90) was smaller than that during the peridiagnosis period (pooled OR = 3.44, 95% CI 1.80–6.57), but remained significant. The results of the subgroup analyses for CD diagnostic method, sample size, and geographic region are shown in Table 2.

### Coeliac Disease and Esophageal Cancer

**FIGURE 4:**
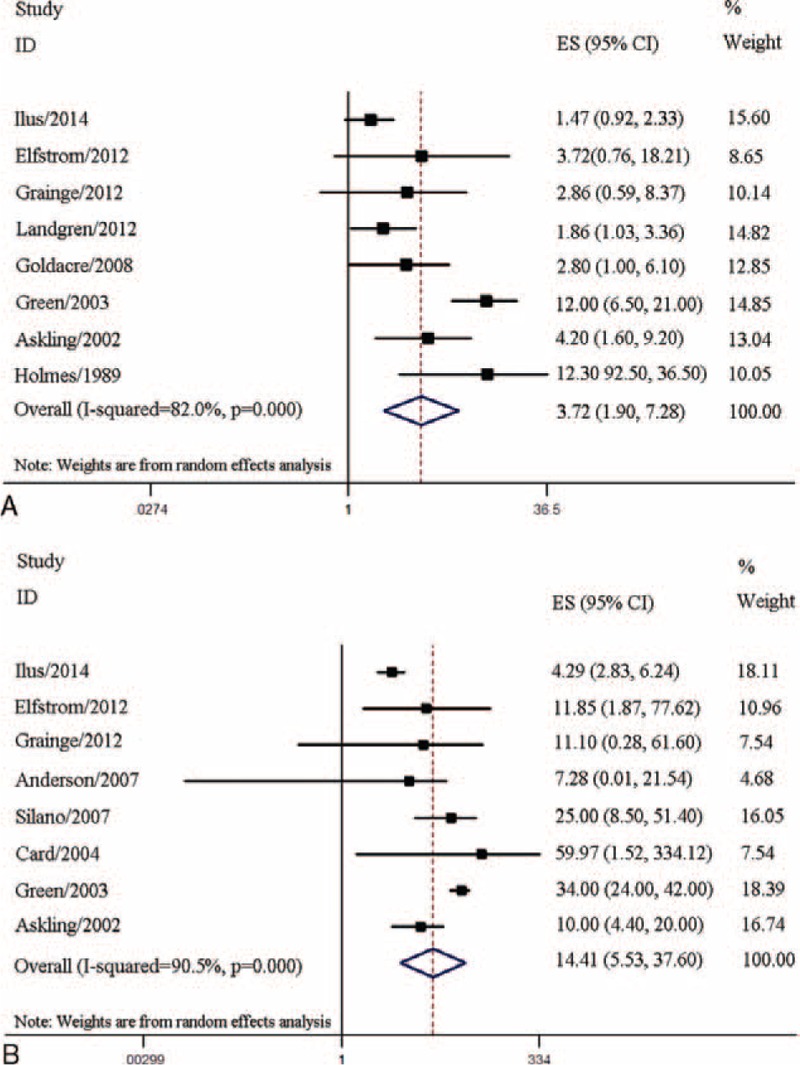
Association between coeliac disease and risks of esophageal and small intestinal cancers. (A) Meta-analysis of coeliac disease and esophageal cancer risk. (B) Meta-analysis of coeliac disease and small intestinal cancer risk.

A total of 8 studies with 79,365 CD patients were included, and 67 esophageal cancer patients were identified. The pooled OR for esophageal cancer was 3.72 (95% CI 1.90–7.28) with significant heterogeneity (I^2^ = 82.0%, *P* < 0.001) (Table 1, Figure 4A), suggesting that CD patients had a higher risk of developing esophageal cancer. Moreover, esophageal cancer risk was higher in the peridiagnosis period (pooled OR = 4.02, 95% CI 1.54–10.52) than in the postdiagnosis period (pooled OR = 2.17, 95% CI 1.34–3.51). Results of the subgroup analyses for study design, comparison population, diagnostic method, sample size, and geographic region are shown in Table 3.

**TABLE 3:**
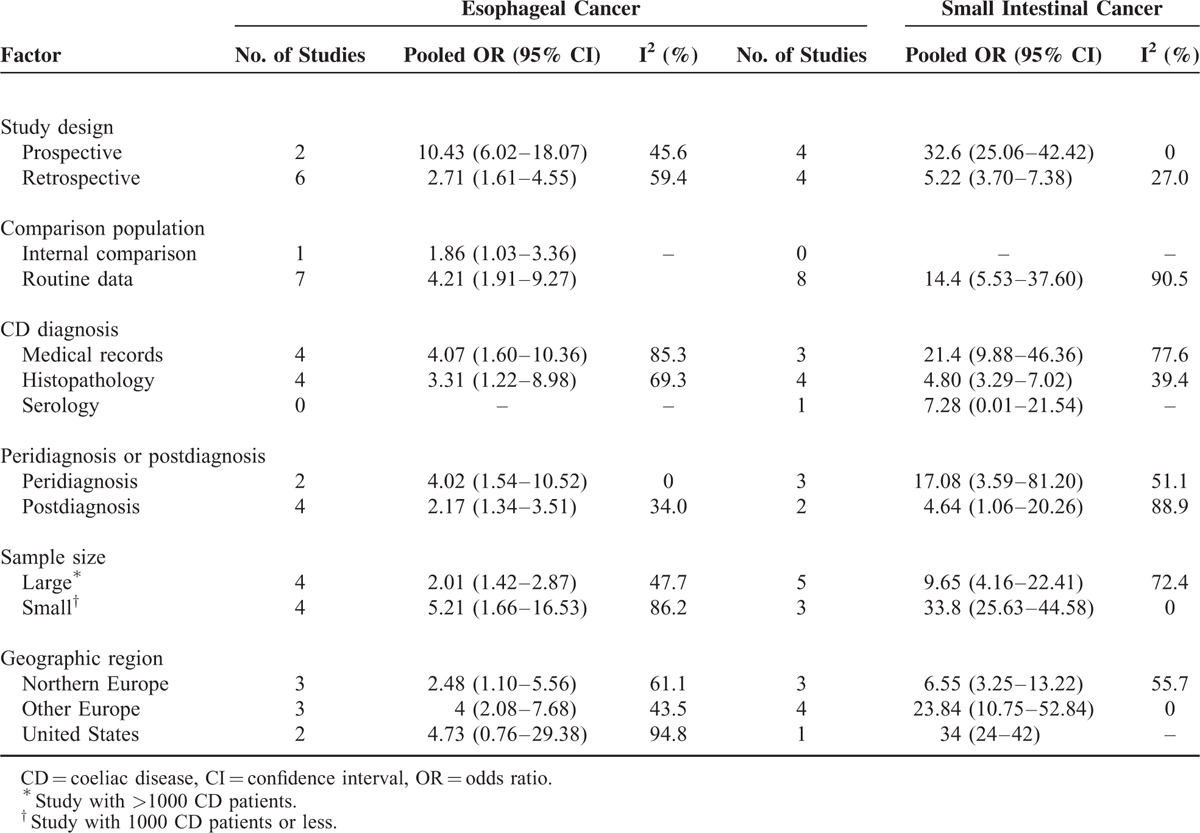
Subgroup Analyses of Risk of Esophageal, Small Intestinal, and Breast Cancer

### Coeliac Disease and Small Intestinal Carcinoma

Eight studies with 79,991 CD patients including 75 small intestinal carcinoma patients were assessed; CD patients were at higher risk of small intestinal carcinoma (pooled OR = 14.41, 95% CI 5.53–37.60) with significant heterogeneity (I^2^ = 90.5%, *P* < 0.001) (Table 1, Figure 4B). Risk of small intestinal carcinoma in the peridiagnosis period (pooled OR = 17.08, 95% CI 3.59–81.20) was higher than that in the postdiagnosis period (pooled OR = 4.64, 95% CI 1.06–20.26). Results of subgroup analyses for study design, comparison population, diagnostic method, sample size, and geographic region are shown in Table 3.

### Other GI Cancers

The pooled results indicated no significant associations between CD and risk of gastric cancer (n = 6, pooled OR = 1.53, 95% CI 0.96–2.44), colon cancer (n = 8, pooled OR = 1.15, 95% CI 0.86–1.56), rectal cancer (n = 5, pooled OR = 0.90, 95% CI 0.71–1.14), liver cancer (n = 4, pooled OR = 2.16, 95% CI 0.94–4.96), or pancreatic cancer (n = 6, pooled OR = 1.51, 95% CI 0.76–2.99) (Table 1).

### Publication Bias

Begg funnel plot and Egger test suggested that publication bias existed in the meta-analysis for assessment of risk of all cancers (*P*_Egger__test_ = 0.01). No publication bias was found for other procedures.

## DISCUSSION

CD is an autoimmune enteropathy that occurs in 1% of the Western population. Although the association between CD and the risk of malignancies has long been investigated, no clear conclusions have been made thus far.^1,7,27,28^ This meta-analysis systematically evaluated this association; the results indicated that CD increased the risks of all malignancies as well as GI malignancy, including esophageal cancer and small intestinal carcinoma, specifically. Compared with the general population, the risk of all malignancies was slightly increased in CD patients (pooled OR = 1.25, 95% CI 1.09–1.44). Subgroup analyses revealed that the risk of all cancers was slightly elevated in both prospective (pooled OR = 1.20, 95% CI 1.02–1.40) and retrospective studies (pooled OR = 1.29, 95% CI 1.08–1.54). Moreover, such association was also observed in the subgroup analyses for sample size and comparison population. Interestingly, we found that CD patients had a higher risk of all cancers in the peridiagnosis period (pooled OR = 1.72, 95% CI 1.14–2.59), but not in the postdiagnosis period (pooled OR = 1.09, 95% CI 0.93–1.27), suggesting a variation in the risk of all cancers with time from diagnosis. A possible explanation is that early symptoms of abdominal cancers are similar to CD symptoms, and the reason for CD diagnosis in some patients is due to the development of cancer.^20^ Moreover, a decrease in the risk of malignancy with time after diagnosis might also be due to the adoption of a gluten-free diet. A study found that a gluten-free diet offers a protective role against malignancy in CD patients, with RRs of 1.2 (*P* > 0.05) for all malignancies in a strict gluten-free diet group and 2.6 (*P* < 0.001) in patients receiving a normal diet or a reduced gluten diet (CIs were not reported).^23^ Moreover, in a study evaluating the risk of colon cancer in CD patients, the SIR was 0.29 (95% CI 0.07–0.45) for all CD patients and 0.07 (95% CI 0.009–0.27) for those receiving a strict gluten-free diet.^9^ However, age at presentation and duration of disease before compliance to a gluten-free diet may also influence malignancy incidence in CD patients. As reported by Silano et al,^4^ patients of older age at CD diagnosis had a higher malignancy risk, suggesting that a gluten-free diet is likely to be inversely associated with risk of malignancies. However, it should be noted that the definition of peri- and postdiagnosis periods differed among the included studies, and studies comparing malignancy risk in gluten-free diets versus normal diets were limited. Thus, more studies, especially prospective population-based cohort studies, are warranted to assess further the risk of malignancy in the peri- and postdiagnosis periods and to evaluate the effects of a gluten-free diet against malignancy. Nevertheless, the overall incidence of all malignancies in CD patients was approximately 1%, which is quite rare.^7^ Thus, the benefits and risks of routine examination for all cancers in CD patients should be investigated further.

For GI cancers specifically, the pooled analysis demonstrated that CD was associated with a 60% increase in GI cancer risk (pooled OR = 1.60, 95% CI 1.39–1.84). Moreover, the risk of GI cancer in CD patients during the postdiagnosis period (pooled OR = 1.49, 95% CI 1.18–1.90) was lower than that during the peridiagnosis period (pooled OR = 3.44, 95% CI 1.80–6.57); however, it remained significant. The pooled OR for esophageal cancer was 3.72 (95% CI 1.90–7.28), suggesting that CD patients have a higher risk of developing esophageal cancer. Previous literature suggests that CD might be associated with esophageal dysmotility, chronic gastroesophageal reflux, and subsequent chronic esophagitis,^8,20,23,29,30^ and these autoimmune conditions might contribute to esophageal cancer.^24^ The current study also showed that CD patients were at higher risk of small intestinal carcinoma (pooled OR = 14.41, 95% CI 5.53–37.60). Small intestinal carcinoma is a major cause of mortality in young adult patients with early onset CD and is triggered by chronic intestinal mucosal inflammation.^31^ In previous studies, mucosal lesions were located mainly in the proximal small bowel, particularly in the ileum, and CD-mediated small intestinal carcinoma followed a similar distribution.^32,33^ Palascak-Juif et al^34^ suggested that prophylactic surgery (in most cases, ileal resections) would prevent 70% of small intestinal carcinoma if performed after 10 years of follow-up. However, the necessity of ileal resection requires further evaluation. It is hypothesized that gluten-free diets are also fiber-less diets, which may alter intestinal microbiota and subsequently affect cancer risk in CD patients.^35,36^ However, these hypotheses lack sufficient evidence, and further research is warranted to assess the relationship between gluten-free diets and intestinal microbiota alterations. No significant associations were found in the analysis of colon and rectal cancers (colon cancer, pooled OR = 1.15, 95% CI 0.86–1.56; rectal cancer, pooled OR = 0.90, 95% CI 0.71–1.14). Moreover, pooled analyses indicated null significant associations between CD and risks of gastric cancer (pooled OR = 1.53, 95% CI 0.96–2.44), liver cancer (pooled OR = 2.16, 95% CI 0.94–4.96), and pancreatic cancer (pooled OR = 1.51, 95% CI 0.76–2.99).

This study has several limitations. First, the controls were not uniformly defined and most of the included studies used routine data as control groups; observational studies using internal control are considered more accurate than those using external sources of control.^21^ We conducted subgroup analyses according to control groups to solve this problem partially. Second, the number of studies included in some subgroup analyses was relatively small, which may have resulted in less accurate estimates. Moreover, this meta-analysis was not able to control for confounding factors in the included studies, which may have resulted in biased pooled results. Therefore, more precise studies are warranted. It should also be noted that all included studies were conducted in Europe and the United States; thus, the results should be considered with caution for other populations.

This meta-analysis systematically evaluated the association between CD and risk of all cancers as well as risk of GI cancer specifically. We conclude that the overall incidence of malignancy was slightly increased in CD patients, as was the risk of GI cancer, owing to a higher risk of esophageal and small intestinal cancers in CD patients. Additional well-designed studies, especially prospective population-based cohort studies using internal controls, are warranted.
